# 2-(4-Hydroxy-3-methoxyphenyl)-benzothiazole suppresses tumor progression and metastatic potential of breast cancer cells by inducing ubiquitin ligase CHIP

**DOI:** 10.1038/srep07095

**Published:** 2014-11-18

**Authors:** Hiromi Hiyoshi, Natsuka Goto, Mai Tsuchiya, Keisuke Iida, Yuka Nakajima, Naoya Hirata, Yasunari Kanda, Kazuo Nagasawa, Junn Yanagisawa

**Affiliations:** 1 Graduate School of Life and Environmental Sciences, University of Tsukuba, 1-1-1 Tennodai, Tsukuba 305-8577, Japan; 2 Center for Tsukuba Advanced Research Alliance, University of Tsukuba, 1-1-1 Tennodai, Tsukuba 305-8577, Japan; 3 Faculty of Technology, Tokyo University of Agriculture and Technology-TUAT, 2-24-16 Naka-cho, Koganei-shi, Tokyo 185-0031, Japan; 4 Division of Pharmacology, National Institute of Health Sciences, 1-18-1, Kamiyoga, Setagaya-ku 158-8501, Japan

## Abstract

Breast cancer is the most common malignancy among women and has poor survival and high recurrence rates for aggressive metastatic disease. Notably, triple-negative breast cancer (TNBC) is a highly aggressive cancer and there is no preferred agent for TNBC therapy. In this study, we show that a novel agent, 2-(4-hydroxy-3-methoxyphenyl)-benzothiazole (YL-109), has ability to inhibit breast cancer cell growth and invasiveness *in vitro* and *in vivo*. In addition, YL-109 repressed the sphere-forming ability and the expression of stem cell markers in MDA-MB-231 mammosphere cultures. YL-109 increased the expression of carboxyl terminus of Hsp70-interacting protein (CHIP), which suppresses tumorigenic and metastatic potential of breast cancer cells by inhibiting the oncogenic pathway. YL-109 induced *CHIP* transcription because of the recruitment of the aryl hydrocarbon receptor (AhR) to upstream of *CHIP* gene in MDA-MB-231 cells. Consistently, the antitumor effects of YL-109 were depressed by *CHIP* or *AhR* knockdown in MDA-MB-231 cells. Taken together, our findings indicate that a novel agent YL-109 inhibits cell growth and metastatic potential by inducing CHIP expression through AhR signaling and reduces cancer stem cell properties in MDA-MB-231 cells. It suggests that YL-109 is a potential candidate for breast cancer therapy.

Breast cancer is the major cause of cancer death among women worldwide. Triple-negative breast cancer (TNBC), which has been reported to represent approximately 15% of all breast cancers^1^, is characterized by the absence of estrogen receptors (ERs), progesterone receptors (PRs), and human epidermal growth factor-2 (HER-2) expression^2^. TNBC is an aggressive cancer, characterized by rapid tumor growth, a high incidence of metastasis, an increased rate of distant recurrence, and a poor prognosis compared with other breast cancer subtypes^3^. Unlike ER/PR-positive or HER-2-overexpressing subtypes, the effective treatment options for TNBC are limited to cytotoxic therapies because of the lack of molecular targets. Moreover, TNBC cells show a profile that is similar to breast cancer stem cells, which have a strong resistance to chemotherapeutic drugs^4^,^5^. Therefore, new therapeutic options and strategies are required for TNBC therapy.

The carboxyl terminus of Hsp70-interacting protein (CHIP, also named STUB1) is a potential target for the treatment of TNBC. CHIP is a U-box-type ubiquitin E3 ligase that induces ubiquitylation and degradation of its substrates. These include several oncogenic proteins that suppress the tumorigenic and metastatic potential of breast cancer cells^6^,^7^,^8^. We previously reported that CHIP levels were much higher in MCF-7 cells, a non-aggressive cell line derived from human breast cancer cells, than in MDA-MB-231 cells, a highly aggressive cell line. Furthermore, CHIP levels are negatively correlated with the malignancy of human breast tumor tissues^9^. In addition, CHIP suppresses both tumor growth and metastasis in a nude mouse xenograft model. Thus, it has been suggested that the regulation of CHIP expression may represent a potential new clinical approach to TNBC therapy.

Aryl hydrocarbon receptor (AhR) has also recently emerged as a potential therapeutic target for breast cancer. The AhR is a basic helix-loop-helix transcription factor that was initially identified as a receptor for environmental toxins, such as dioxin^10^. Ligand binding to the receptor triggers formation of a heterodimeric nuclear AhR complex, which binds to dioxin response elements in target gene promoters to induce transcriptional activation^11^. Several studies have demonstrated that the AhR may be a potential drug target for several diseases, including endometrial, prostate, pancreatic, and ER-positive breast cancers^12^,^13^,^14^,^15^,^16^,^17^. In addition, the antitumor effects of compounds belonging to the 2-(4-amino-3-methylphenyl) benzothiazole group are mediated by AhR in ER-positive breast cancer cells^18^,^19^,^20^. Phortress, the lysine amide prodrug of 2-(4-amino-3-methylphenyl)-5-fluorobenzothiazole, has completed Phase I clinical evaluations^18^,^21^. In addition to 2-(4-aminophenyl) benzothiazoles, the relatively non-toxic selective AhR modulators (SAhRMs) are highly effective agents for inhibiting hormone-responsive breast cancer growth in animal models^17^,^22^. Although 2-(4-aminophenyl) benzothiazoles and SAhRMs are less effective against ER-negative breast cancer cells, AhR is also expressed in these cells^18^,^23^,^24^. Therefore, we hypothesized that ideal agents might exert the antitumor effects mediated by AhR signaling in both ER-positive and -negative breast cancer cells.

In this study, we demonstrated that the novel agent 2-(4-hydroxy-3-methoxyphenyl)-benzothiazole (YL-109) has ability to inhibit breast cancer progression in TNBC, MDA-MB-231 cells, and ER-positive breast cancer MCF-7 cells. In addition, YL-109 suppresses the proliferation and invasiveness of MDA-MB-231 cells, both *in vitro* and *in vivo*. Moreover, YL-109 suppresses the properties of breast cancer stem cells. Furthermore, we demonstrated that YL-109 increases *CHIP* expression by the recruitment of AhR to an upstream region of the gene. Consistent with these observations, CHIP or AhR knockdowns inhibit the suppressive effects of YL-109 on anchorage-independent growth and invasiveness. Taken together, our findings indicate that YL-109 is a novel antitumor agent that can induce CHIP expression through AhR signaling, and that it represents a promising candidate for a new therapeutic strategy against TNBC.

## Results

### YL-109 inhibits cell proliferation, motility, and invasiveness in breast cancer cells

**Figure 1:**
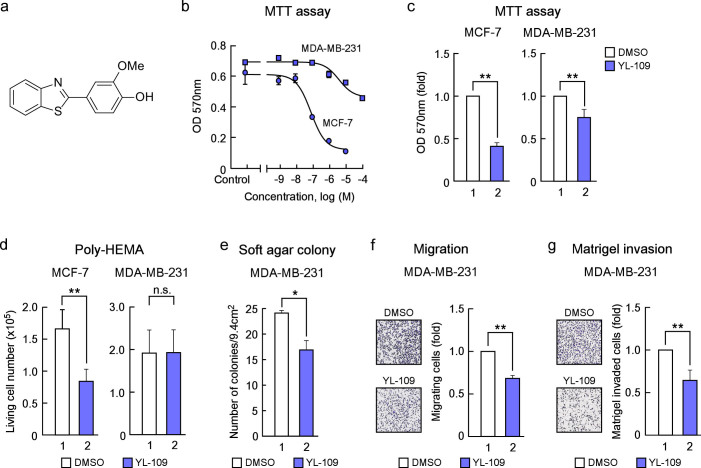
Identification of a compound that inhibits cell proliferation, motility and invasiveness in breast cancer cells. (a) Structure of 2-(4-hydroxy-3-methoxyphenyl)-benzothiazole, YL-109. (b) Effects of YL-109 on cell proliferation in breast cancer cells. MCF-7 or MDA-MB-231 cells were cultured with the indicated concentration (shown as -logM) of YL-109. After 96 h, MTT assay was performed (MCF-7; IC_50_ = 85.8 nM, MDA-MB-231; IC_50_ = 4.02 μM). (c) Effects of YL-109 on anchorage-dependent cell growth in breast cancer cells. MCF-7 or MDA-MB-231 cells were cultured in media containing DMSO or YL-109 (1 μM) for 96 h. The cell viability was measured by MTT assays. (d and e) Effects of YL-109 on anchorage-independent cell growth in breast cancer cells. Cells were plated on poly-HEMA (d) or soft agar (e) coated plates in the absence or presence of YL-109 (1 μM). The viable cells were counted using Countess Automated Cell Counter (Invitrogen) (d). The colonies were examined under a microscope and colonies with a diameter of more than 100 μm were counted (e). (f and g) Effects of YL-109 on cell motility and invasiveness in MDA-MB-231 cells. MDA-MB-231 cells were seeded onto filters with an 8 μm pore size in uncoated (f, migration assay) or Matrigel matrix-coated (g, invasion assay) upper chambers in the absence or presence of YL-109 (1 μM). * indicates p<0.05 and ** indicates p<0.01 and n.s. indicates p>0.05 by student's T test vs. DMSO-treated cells.

It has been reported that 2-(4-aminophenyl)-benzothiazoles have anti-proliferative activity in MCF-7, ER-positive breast cancer cells^18^,^19^,^20^. Therefore, we investigated the effects of 2-(4-hydroxy-3-methoxyphenyl)-benzothiazole, YL-109 (Figure 1a) on cell proliferation in MCF-7 cells. YL-109 possesses characteristic hydroxyl group at C4, whereas 2-(4-aminophenyl)-benzothiazoles have the amino group at this position. YL-109 strongly inhibited cell proliferation of MCF-7 cells in a dose-dependent manner (IC_50_ = 85.8 nM) (Figure 1b and c). Surprisingly, YL-109 had an anti-proliferative effect in a dose-dependent manner (IC_50_ = 4.02 μM) on MDA-MB-231 cells, known as TNBC cells, unlike 2-(4-aminophenyl)-benzothiazoles (Figure 1b and c)^18^. We subsequently tested whether YL-109 inhibited anchorage-independent growth in poly-HEMA coated plates and colony formation in soft agar (Figure 1d and e). Under short-term detached conditions using poly-HEMA coated plate, YL-109 suppressed cell survival in MCF-7 cells, but not in MDA-MB-231 cells (Figure 1d). In contrast, YL-109 decreased the number of colonies in MDA-MB-231 cells under long-term detached conditions on soft agar (Figure 1e). Moreover, to examine the effects of YL-109 on the metastatic and invasive potential of MDA-MB-231 cells, we performed migration and invasion assays. In the migration assay, YL-109 reduced the ability of cells to migrate (Figure 1f). YL-109 also significantly decreased the number of cells that penetrated the Matrigel-coated membrane (Figure 1g). In addition, YL-109 inhibited cell proliferation and invasiveness of BT-20 cells, also known as TNBC cells (see Supplementary Fig. S1 online). These results suggest that YL-109 inhibits cell proliferation and suppresses the metastatic potential of breast cancer cells.

### YL-109 inhibits both tumor growth and cancer metastasis of breast cancer cells *in vivo*

**Figure 2:**
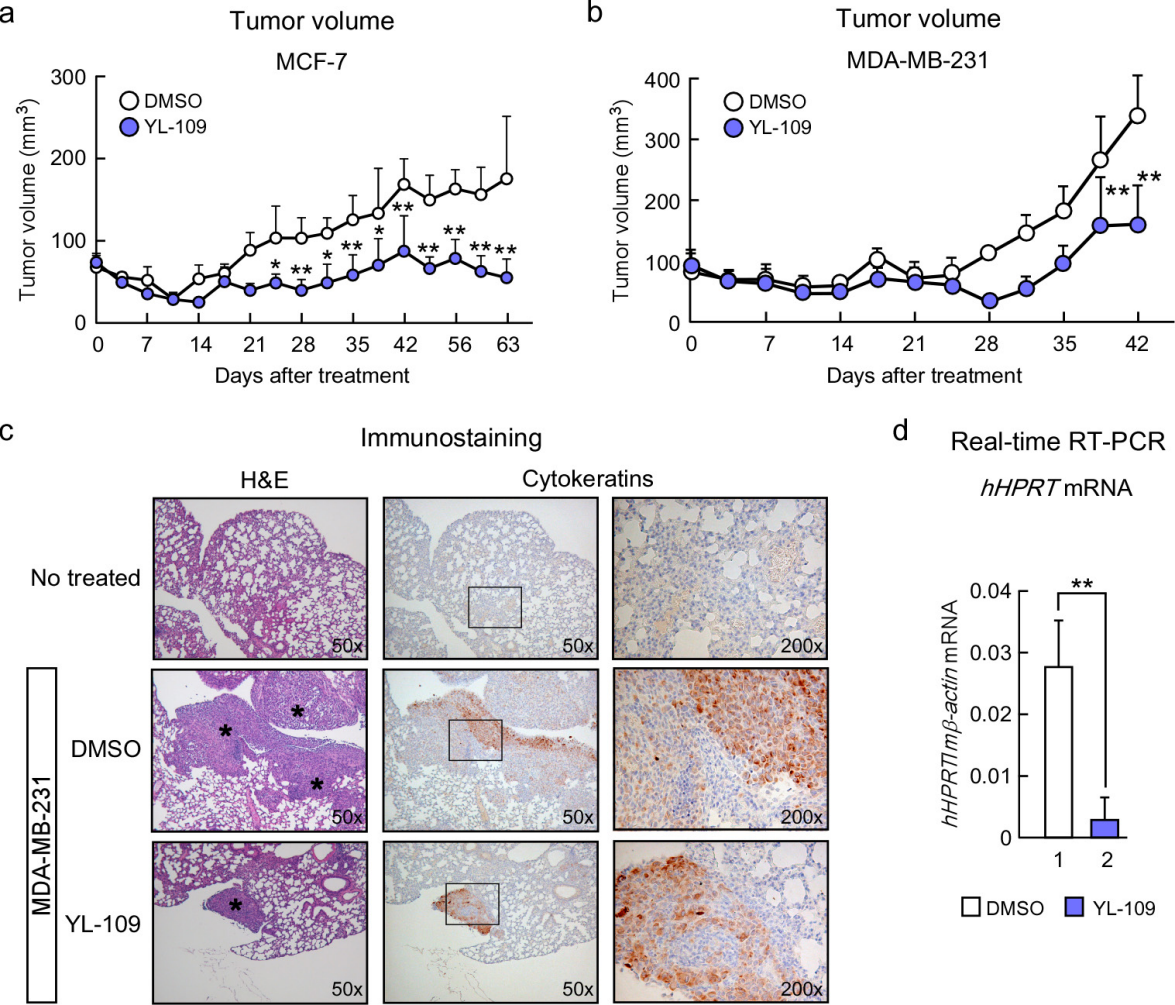
YL-109 suppresses both tumor growth and metastasis of breast cancer cells *in vivo*. (a and b) Effects of YL-109 on tumor growth in a mouse xenograft model. Mice were treated with DMSO (vehicle) or YL-109 (15 mg/kg) for every 2 days. Tumor growth curves showed tumor volume in nude mice inoculated with MCF-7 (a) or MDA-MB-231 cells (b). Tumor growth was monitored twice each week. Bars represent mean + s.d. (n = 3–6). * indicates p<0.05 and ** indicates p<0.01 by two-way ANOVA with Bonferroni's post hoc test. (c and d) Effects of YL-109 on tumor metastasis *in vivo*. MDA-MB-231 cells were injected into the tail veins of nude mice. Forty-two days after the injections, lungs were collected. Representative images of sections from lungs are shown (c). Left panels show the images of H&E staining (50x). Immunohistochemistry for human cytokeratins is shown in middle (50x) and right (200x) panels. Asterisks indicate metastatic tumor growth. The lung metastasis was quantified by real-time RT–PCR (d). Specific primers for human *HPRT* were used. * indicates p<0.05 and ** indicates p<0.01 by student's T test vs. DMSO-treated mice.

Using a nude mouse xenograft model, we investigated the effects of YL-109 *in vivo*. Mice treated with vehicle showed significantly enlarged tumors, whereas mice treated with YL-109 showed attenuated tumor growth using MCF-7 cells (Figure 2a). Interestingly, YL-109 also suppressed tumor growth in mice injected with MDA-MB-231 cells (Figure 2b). Next, to examine the effect of YL-109 against metastatic activity, we performed an *in vivo* lung metastasis assay using MDA-MB-231 cells. We discovered several metastatic tumors in the lungs of mice injected with MDA-MB-231 cells in the vehicle (Figure 2c). Compared with the vehicle control, YL-109 significantly reduced lung metastasis (Figure 2c). We also quantified lung metastasis using real-time RT-PCR, which confirmed our observations (Figure 2d). These results suggest that YL-109 suppresses tumor progression of breast cancer cells *in vivo*.

### YL-109 suppresses breast cancer progression by inducing CHIP expression

**Figure 3:**
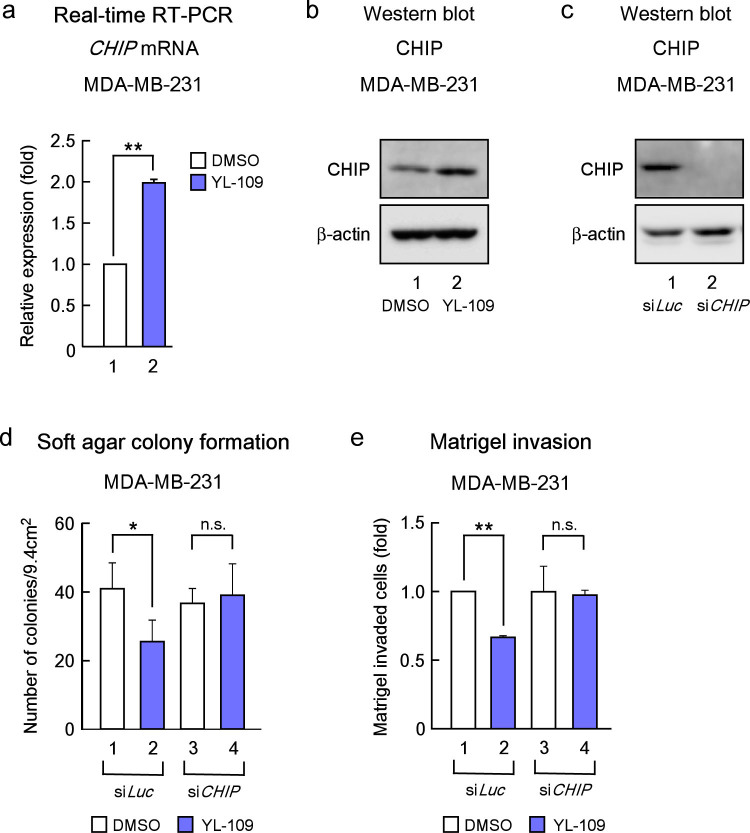
YL-109 inhibits breast cancer progression by inducing CHIP expression. (a and b) Effect of YL-109 on CHIP levels in MDA-MB-231 cells. MDA-MB-231 cells were cultured in the absence or presence of YL-109 (1 μM). Total RNA was prepared from the indicated cells and the expression of *CHIP* was analyzed using real-time RT–PCR (a). Protein levels of CHIP were determined by Western blotting (b). Full length images of blots are represented in Supplementary Fig. S2. (c) Knock-down of CHIP by treatment with siRNA targeting CHIP. The levels of CHIP were examined by western blotting. Full length images of blots are represented in Supplementary Fig. S2. (d) Effects of CHIP knockdown on YL-109-induced reduction of anchorage-independent cell growth in MDA-MB-231 cells. CHIP expression was knocked down in MDA-MB-231 cells, and cells were plated in soft agar dishes in the absence or presence of YL-109 (1 μM). After incubation for 3 weeks, colonies were examined under a microscope and colonies with a diameter of more than 50 μm were counted. (e) Effects of CHIP knockdown on YL-109-reduced invasiveness in MDA-MB-231 cells. MDA-MB-231 cells were knocked down of CHIP and pre-cultured in the absence or presence of YL-109 (1 μM) for 48 h. The cells were seeded onto filters with Matrigel matrix-coated upper chambers in the absence or presence of YL-109 (1 μM). After 24 h incubation, invaded cells were stained using crystal violet. * indicates p<0.05, ** indicates p<0.01, and n.s. indicates p>0.05 by student's T test vs. DMSO-treated cells.

We previously demonstrated that CHIP suppresses tumorigenesis and the metastatic cellular phenotypes of breast cancer cells both *in vitro* and *in vivo*^9^. It has also been indicated that CHIP expression is significantly associated with prognostic parameters in breast cancer patients^25^. Therefore, we examined whether YL-109 induced CHIP expression. We observed that YL-109 increased both CHIP mRNA and protein levels (Figure 3a and b). Next, to investigate whether the antitumor effects of YL-109 were mediated by CHIP, we performed colony formation and Matrigel invasion assay using siRNA for CHIP (Figure 3c–e). YL-109-induced inhibition of cell growth and invasive potential were decreased by CHIP knockdown (Figure 3d and e). These results demonstrate that YL-109 inhibits breast cancer progression by inducing CHIP expression.

### YL-109 activates AhR signaling to induce CHIP expression

**Figure 4:**
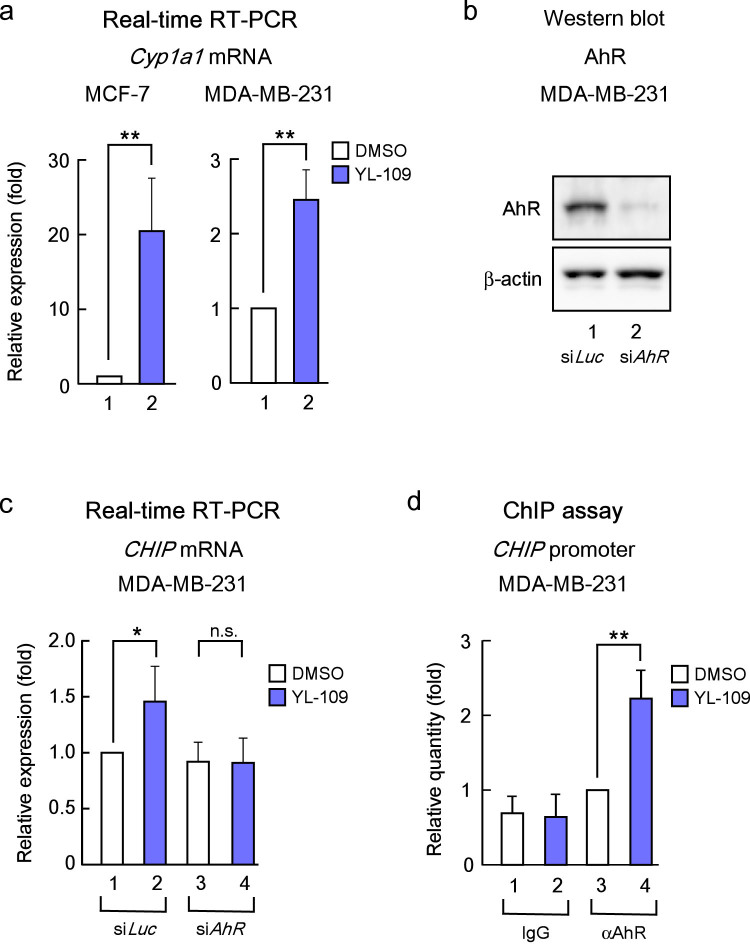
YL-109 induces CHIP expression through AhR activation. (a) Effects of YL-109 on *Cyp1a1* mRNA expression in breast cancer cells. MCF-7 or MDA-MB-231 cells were cultured in the absence or presence of YL-109 (1 μM). *Cyp1a1* mRNA level was quantified using real-time RT-PCRs. (b) Knock-down of AhR by treatment with siRNA targeting AhR. Western blotting was used to examine the levels of AhR. Full length images of blots are represented in Supplementary Fig. S2. The representative blots for AhR were cropped to clarify relevant bands. (c) Effects of AhR knockdown on YL-109-increased *CHIP* expression. AhR expression was knocked down in MDA-MB-231 cells, and cells were cultured in the absence or presence of YL-109 (1 μM). *CHIP* expression was analyzed using real-time RT-PCRs. (d) AhR recruitment to *CHIP* promoter by YL-109. MDA-MB-231 cells were cultured in the absence or presence of YL-109 (1 μM). ChIP assay was performed with control IgG or anti-AhR antibodies. Immunoprecipitated DNA was examined using real-time RT-PCR and primers specific for the *CHIP* promoter. Samples were normalized to the amount of input DNA. * indicates p<0.05, ** indicates p<0.01, and n.s. indicates p>0.05 by student's T test vs. DMSO-treated cells.

Previous studies indicated that 2-(4-aminophenyl)-benzothiazoles activate AhR and increase transcription of AhR target genes, such as *Cyp1a1*^18^,^19^,^20^. We observed that YL-109 induced *Cyp1a1* expression in MCF-7 and MDA-MB-231 cells (Figure 4a). To explore AhR participation in the mechanisms underlying the induction of CHIP by YL-109, we examined whether CHIP expression levels were affected by knocking down of AhR with siRNA (Figure 4b and c). We observed that YL-109-induced increases in *CHIP* mRNA were reduced by AhR knockdown (Figure 4c). We subsequently examined AhR recruitment to the promoter region in *CHIP* gene, which contains potential AhR response elements. Chromatin immunoprecipitation (ChIP) assay demonstrated that the interaction between AhR and the *CHIP* promoter is potentiated by YL-109 (Figure 4d). Our data suggest that YL-109 induces CHIP expression through recruitment of AhR to upstream of *CHIP* gene.

### YL-109 exerts antitumor effects through AhR signaling

**Figure 5:**
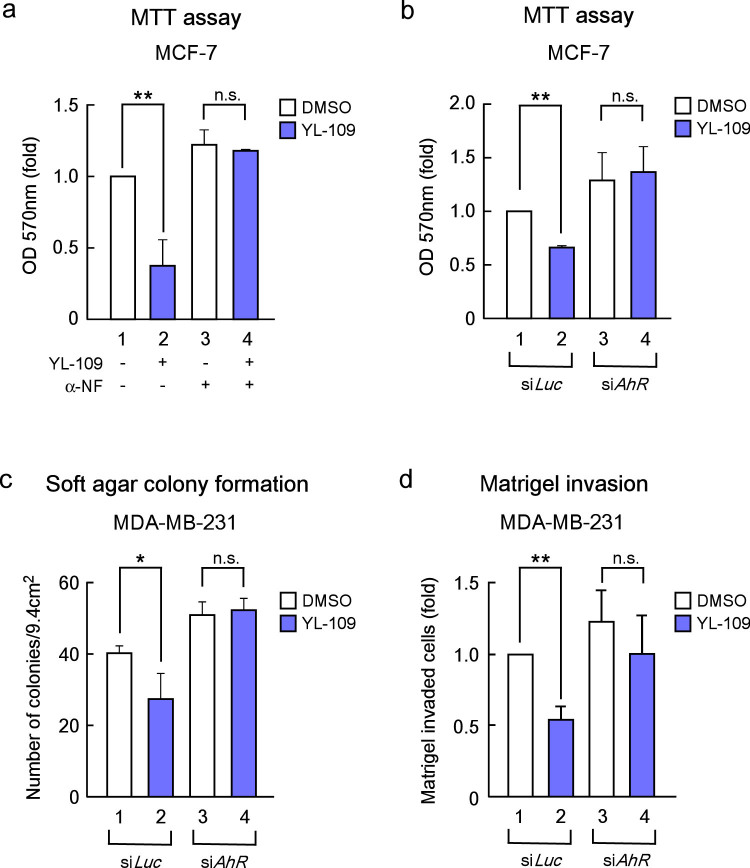
YL-109 suppresses breast cancer progression through AhR signaling. (a and b) Effects of AhR on YL-109-inhibited cell proliferation in MCF-7 cells. MCF-7 cells were cultured in the absence or presence of YL-109 (1 μM), AhR antagonist α-NF (1 μM) (a). MCF-7 cells knocked down of AhR were cultured in the absence or presence of YL-109 (1 μM) (b). The cell viability was measured by MTT assays. (c) Effects of AhR knockdown on YL-109-inhibited anchorage-independent cell growth in MDA-MB-231 cells. AhR expression was knocked down in MDA-MB-231 cells, and cells were plated in soft agar dishes in the absence or presence of YL-109 (1 μM). After incubation for 3 weeks, colonies were examined under a microscope and colonies with a diameter of more than 50 μm were counted. (d) Effects of AhR knockdown on YL-109-reduced invasiveness in MDA-MB-231 cells. MDA-MB-231 cells were seeded onto filters with Matrigel matrix-coated upper chambers in the absence or presence of YL-109 (1 μM). * indicates p<0.05, ** indicates p<0.01, and n.s. indicates p>0.05 by student's T test vs. DMSO-treated cells.

To examine whether AhR signaling mediated the inhibition of cell proliferation by YL-109, we performed MTT assay using the AhR antagonist α-naphtoflavone (α-NF) and siRNA against AhR (Figure 5a and b). Treatment with α-NF and knockdown of AhR expression abolished the inhibitory effect of YL-109 on cell proliferation of MCF-7 cells (Figure 5a and b). Moreover, YL-109-induced inhibition of colony formation and invasive potential were repressed by AhR knockdown (Figure 5c and d). Our data suggest that the antitumor effects of YL-109 are mediated by AhR signaling.

### YL-109 reduces the property of cancer stem cells in breast cancer cells

**Figure 6:**
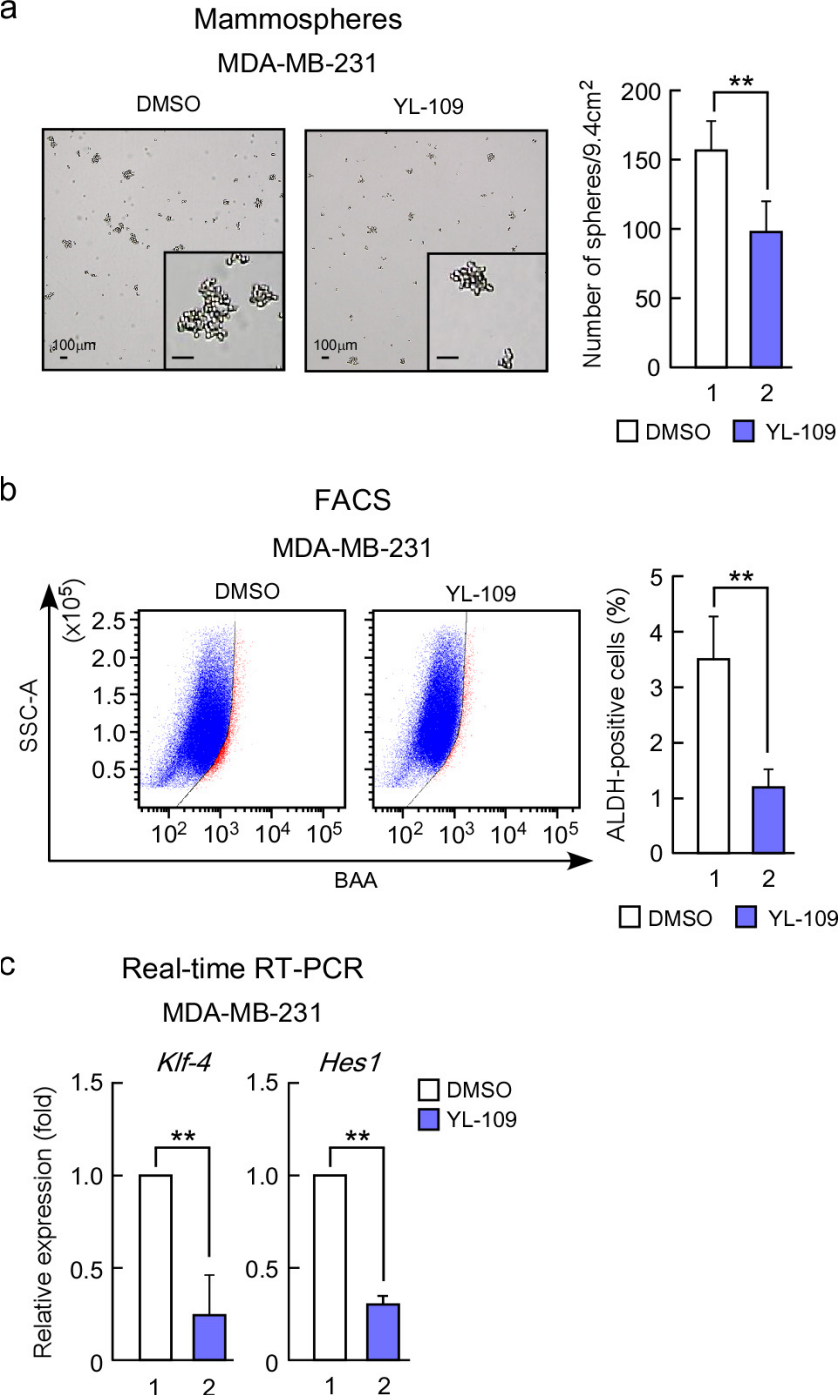
YL-109 reduces cancer stem cell properties in MDA-MB-231 cells. (a) Effects of YL-109 on Mammosphere formation. MDA-MB-231 cells were inoculated onto ultra-low-attachment plates in the absence or presence of YL-109 (1 μM. After 7 days, mammospheres with a diameter of more than 100 μm were counted. The photographs in the left panel show representative mammospheres. The insets in panels show magnified images. The scale bars represent 100 μm. The graph in right panel shows the number of mammospheres. (b) Effects of YL-109 on ALDH-positive cell population. MDA-MB-231 cells were incubated with YL-109 (1 μM) for 96 h and ALDH-positive cell population was measured by the Aldefluor assay kit and flow cytometry. (c) Effects of YL-109 on the expression of cancer stem cell markers in mammospheres. The mammospheres were collected after 7 days culture on ultra-low-attachment plates in the absence or presence of YL-109 (1 μM). The gene expressions of *Klf-4* and *Hes1* were analyzed using real-time RT-PCR. ** indicates p<0.01 by student's T test vs. DMSO-treated cells.

Recently, we have shown that CHIP suppresses cancer stem cell properties in breast cancer cells^26^. Therefore, we evaluated whether YL-109 affects breast cancer stem cells. One property of cancer stem cells is their ability to form tumor spheres, denoted mammospheres in the case of breast cancer^27^. We observed that cells derived from the MDA-MB-231 cell line formed mammospheres, whereas YL-109 markedly inhibited mammosphere formation (Figure 6a). To confirm the effects of YL-109 on the population of cancer stem cells in MDA-MB-231 cells, we used flow cytometry with cancer stem cell marker ALDH. Consistent with the effects on mammosphere formation, YL-109 decreased ALDH-positive cell population (Figure 6b). In addition, YL-109 significantly decreased mRNA levels of *Klf-4* and Notch target gene *Hes1* in MDA-MB-231 mammosphere cultures (Figure 6c). Taken together, these results indicate that YL-109 inhibits the properties of breast cancer stem cells.

## Discussion

In the present study, we revealed that the novel agent YL-109 has antitumor activity in TNBC cells. It was previously reported that 2-(4-aminophenyl)-benzothiazoles have anti-proliferative activity in ER-positive breast cancer cells, that is mediated by the AhR signaling pathway. AhR is expressed in breast cancer cells regardless of ER expression^23^,^24^. However, TNBC cells exhibit a poor response to benzothiazoles and SAhRMs because they do not activate AhR signaling^18^. Consistent with previous reports on benzothiazole antitumor activity, YL-109 inhibited cell proliferation through AhR activation in ER-positive breast cancer cells. In addition, we observed that YL-109 could activate AhR signaling in MDA-MB-231 cells, which is necessary for YL-109 to exert antitumor effects in these cells. These results suggest that YL-109 inhibits breast cancer progression by AhR signaling activation in TNBC cells.

Our results also demonstrated that the YL-109-induced AhR signaling activation resulted in increased CHIP expression. We previously reported that CHIP suppresses tumor progression in human breast cancer by inhibiting oncogenic pathways^9^. In mice, tumor growth and metastasis were significantly inhibited by CHIP expression, whereas CHIP knockdowns in breast cancer cells resulted in rapid tumor growth and metastatic phenotypes. In this study, we observed that YL-109 treatment increased CHIP expression and CHIP knockdown inhibited the suppressive effects of YL-109 on both breast cancer cell growth and invasiveness. Moreover, YL-109 induced CHIP expression by recruiting AhR to an upstream region of the *CHIP* gene. We also found that the multiple AhR-responsive element sites exist in the promoter region of *CHIP*, which contain the core sequence 5′-GCGTG-3′. Taken together, these findings indicate that YL-109 inhibits breast cancer progression by inducing CHIP expression through AhR signaling.

Our data demonstrated that YL-109 increased CHIP levels in MDA-MB-231 cells. We also observed that YL-109 inhibited anchorage-independent cell growth in soft agar as well as xenograft tumor growth, but not in poly-HEMA coated plates. For assays of *in vitro* cell growth, cells were detached and suspended, either in poly-HEMA coated plates for 24 h, or in soft agar for 3 weeks. In short-term growth experiments such as the poly-HEMA assay, we did not observe cell growth inhibition by YL-109, because YL-109-induced inhibition of cell growth presumably requires the elevation of CHIP levels. Consistent with this, in long-term growth experiments such as soft agar colony formation and xenograft tumor growth, YL-109 inhibited cell growth of TNBC cells. Furthermore, this effect was repressed by the knockdown of CHIP or AhR in TNBC cells.

In contrast, CHIP levels are considerably higher in MCF-7 cells than in MDA-MB-231 cells, and we observed that YL-109 did not affect CHIP expression in MCF-7 cells (data not shown). However, YL-109 was able to inhibit the growth of MCF-7 cells in both short-term and long-term growth experiments. These observations suggest that CHIP does not contribute to the effects of YL-109 on cell growth in ER-positive breast cancer cells.

Previous studies have reported that the inhibition of ER signaling can result from cross talk with the ligand-activated AhR. AhR ligands strongly suppress estrogen-induced responses in the rodent uterus, mammary tumors, and human breast cancer cells^28^. Treatment of ER-positive breast cancer cells with 2,3,7,8-tetrachlorodeibenzo-p-dioxin (TCDD) induces proteasome-dependent degradation of endogenous ERα^29^. In this study, we demonstrated that YL-109 inhibits cell proliferation through the AhR signaling pathway in MCF-7 cells. These findings suggest that YL-109 may induce ERα degradation because of AhR activation in ER-positive breast cancer cells. Taken together, our results show that YL-109 suppresses cell growth through AhR signaling in both ER-positive and triple-negative breast cancer cells. In TNBC cells, YL-109 increases CHIP levels by AhR activation. In contrast, in ER-positive cells, YL-109 induces ERα degradation by AhR activation.

The treatment options for patients with TNBC, including those with ER-negative breast cancer, have not yet been established. In this study, we have demonstrated that YL-109 has antitumor activity in TNBC cells, and thus represents a potential TNBC therapy. In addition, resistance to anti-estrogens like tamoxifen is a major clinical problem in the treatment of hormone-dependent breast cancer including ER-positive breast cancer. Our results indicated that YL-109 inhibited tumor growth using a different mechanism from anti-estrogens in ER-positive breast cancer cells. Therefore, we predict that YL-109 will also exert an antitumor effect in tamoxifen-resistant breast cancer. Thus, YL-109 might have the ability to treat with several subtypes of breast cancer.

Drug resistance is a serious problem in breast cancer therapy, and cancer stem cells contributed to chemotherapeutic drug and radiation resistance^30^. Breast cancer stem cells form mammospheres and express stem cell markers. We observed that YL-109 markedly inhibited mammosphere formation and decreased the ALDH-positive cell population in MDA-MB-231 cells. Moreover, YL-109 significantly decreased *Klf-4* and *Hes1* expression in mammospheres derived from MDA-MB-231 cells. KLF-4 is essential for the maintenance of breast cancer stem cells and cell invasiveness^31^, and Hes1 is a target of Notch signaling, which may be important for the self-renewal of breast cancer stem cells^32^,^33^. Therefore, our results suggest that YL-109 inhibits the properties of breast cancer stem cells and can be used for patients with drug-resistant cancer.

In summary, we demonstrated that YL-109 is a novel antitumor agent that suppresses tumor progression in TNBC cells and inhibits cancer stem cell properties, unlike other benzothiazoles. Furthermore, we show that YL-109 induces CHIP expression through AhR signaling. Taken together, we propose a new therapeutic strategy for breast cancer including TNBC, and prodrug of YL-109 improved water solubility and chemically stability may be helpful in development of new pharmacological treatments for TNBC.

## Methods

### Cell culture

MCF-7, MDA-MB-231 and BT-20 cells were maintained in Dulbecco's modified Eagle's medium (DMEM) or RPMI1640 supplemented with 10% fetal bovine serum (FBS). For the experiments, cells were seeded in phenol red-free DMEM containing 4% charcoal-stripped FBS. After 24 h, the medium was exchanged for phenol red-free DMEM containing 1% charcoal-stripped FBS with or without ligands.

### RNA interference

For transfection of siRNAs, cells at 30–50% confluency were transfected with 20 nmol/L of siRNA using Lipofectamine RNAi max (Invitrogen) according to the manufacture's protocol. All siRNAs were purchased from Invitrogen. The siRNA duplexes

CHIP, 5′-CCAGCGCUCUUCGAAUCGCGAAGAA-3′;

AhR, 5′-GAGAAUUCUUAUUACAGGCUCUGAA-3′.

Stealth^TM^ RNAi reporter control was used as a negative control.

### MTT assay

Cells were incubated in DMEM containing 4% charcoal-stripped FBS with DMSO or YL-109 (1 μM) for 96 h. The proliferation of cultured cells was measured by MTT assay using the MTT Cell Count Kit (Nakarai tesque).

### Poly-HEMA

One gram of poly-(2-hydroxyethyl methacrylate) (poly-HEMA) (Sigma-Aldrich) was dissolved in 25 mL 99.5% ethanol and mixed overnight at 37°C. The poly-HEMA stock solution were added to 12-well plates and plates were left to dry for a few hours. After drying, the plates were washed with PBS. Cells were plated in the poly-HEMA-coated 12-well plates at a density of 1 × 10^5^ cells (MCF-7) or 1.5 × 10^5^ cells (MDA-MB-231) per well and incubated for 24 h. Cells were treated with 0.2% trypan blue. Viable cells were counted using Countess Automated Cell Counter (Invitrogen).

### Soft agar colony-formation assay

For soft agar assays, 2 × 10^5^ cells were suspended in DMEM containing 0.35% agar and layered on top of 1 mL of DMEM solidified with 0.6% agar in each well of a 6-well plate. After growing at 37°C for 3 weeks, colonies were counted under the microscope. The reported results represent the averages of three independent experiments.

### Invasion and migration assay

The invasive potentials of MDA-MB-231 and BT-20 cells were tested with Matrigel invasion chambers (24-well format, 8 μm pore size; BD Biosciences). After incubation in DMEM containing 1% charcoal-stripped FBS with DMSO or YL-109 (1 μM) for 48 h, suspensions (0.5 mL) containing 1 × 10^5^ cells (MDA-MB-231) or 0.5 × 10^5^ cells (BT-20) were added with vehicle alone (DMSO) or YL-109 (1 μM), and transferred into insert chambers. These cells were then incubated for 24 h at 37°C with 0.75 mL of DMEM containing 4% charcoal-stripped FBS and each ligand in the bottom chambers. After incubation, the cells on the upper surface of the filter were removed, and invading cells were fixed in methanol. Fixed cells were stained with crystal violet and counted under a microscope. Migration assays were performed using the same procedure, except that the insert chambers were not coated with Matrigel and cells in chamber were incubated for 12 h.

### Tumor xenograft models

BALB/cAjcl-nu/nu female mice at 4-5 weeks of age were purchased from CLEA Japan. The mice were kept in a pathogen-free environment under controlled conditions of light and humidity. MCF-7 or MDA-MB-231 cells were cultured as monolayers, trypsinized and resuspend in Matrigel (BD Biosciences) at each 1 × 10^8^ or 1 × 10^7^ cells/ml. Each mouse was injected subcutaneously with 100 μL of cell suspension (1 × 10^7^ or 1 × 10^6^ cells) in both flanks. YL-109 was subcutaneously injected in the scruff of the neck (15 mg/kg) for every 2 days. Tumor growth was monitored twice each week by measuring the tumor size using calipers; tumor volume was determined using the formula V = 1/2 × larger diameter × (smaller diameter)^2^. All animal experiments were performed in accordance with institutional guidelines.

### In vivo lung metastasis analysis

BALB/cAjcl-nu/nu female mice at 4-5 weeks of age were purchased from CLEA Japan. The tail vein of each mouse was injected with MDA-MB-231 cells (5 × 10^5^ cells). Forty-two days after injection, the mice were sacrificed and lung metastases were examined by hematoxylin-eosin (H&E) staining and immunohistochemistry for human cytokeratin and quantified by real-time RT-PCRs using primers specific for human *HPRT* that did not cross-react with the mouse homologue. Mouse β-actin was used for normalization.

### Immunohistochemistry

Immunohistochemistry was performed under contract with Genostaff Co., Ltd. Tissues were fixed, dehydrated and embedded in paraffin were cut at 5 μm thickness. For antigen retrieval, the sections were incubated with EDTA buffer [10 mM Tris-HCl and 1 mM ethylenediaminetetraacetic acid (EDTA)] (pH 9.0) at 95°C for 20 min, and then treated with 0.3% hydrogen peroxide. After blocking, the sections were incubated with anti-human-cytokeratin clone MNF-116 (Abcam) (10 μg/ml, overnight at 4°C). Immunostaining was performed with EnVision^TM^ system (Dako). Staining of H&E was performed by standard protocol.

### Real-time RT-PCR

Tissues and cells were homogenized in 1 mL of Sepazol and total RNA was extracted according to the manufacturer's instructions (Nacalai tesque). cDNA was synthesized from total RNA using RevaTraAce reverse transcriptase (Toyobo) and oligo dT primer. Real-time PCRs were performed to amplify fragments representing for the indicated mRNA expression using the Thermal Cycle DiceTM TP800 (Takara) and SYBR Premix Ex Taq (Takara). The primer sequences can be found in Supplementary Table S1.

### Western blotting

Cells were lysed in TNE buffer [10 mM Tris-HCl (pH 7.8), 1% Nonidet P-40 (NP-40), 0.15 M NaCl, and 1 mM EDTA], and then immunoblotted with the appopriate antibodies. A murine hybridoma monoclonal antibody against human CHIP was generated in our laboratory. The antibodies used in this study were: anti- human CHIP (1:500; Green Space Biomed, Japan), AhR (1:500; Santa Cruz), and β-actin (1:5000; Sigma) antibodies. Specific proteins were visualized using an enhanced chemiluminescence (ECL) Western blot detection system (Millipore).

### Chromatin immunoprecipitation (ChIP) assay

This was done essentially as described previously^34^. The DNA was amplified by real-time PCR as described above. The primers for real-time PCR are as follows: forward primer: 5′-TCACATGCTTCTCTGCTCTG-3′; reverse primer: 5′-GACTGTTGGTAGAGTGGAAG-3′ for *CHIP* gene upstream region. Samples were normalized based on the amount of input DNA.

### Mammosphere formation assay

MDA-MB-231 cells were plated onto 6-well ultra-low-attachment plates (Corning Costar) at 5 × 10^3^ cells per well. Cells were maintained in serum-free with a CnT-27 medium and growth additives (CellnTEC Advanced cell systems)^27^. After 7 days, over 100 μm spheres were counted.

### ALDH assays

The ALDEFLUOR kit (Stem Cell Technologies, Durham, NC, USA) was used to detect cancer stem cell population with high aldehyde dehydrogenase (ALDH) enzyme activity, as previously reported^35^. Briefly, MDA-MB-231 cells were treated with YL-109 (1 μM) for 4 days. The cells were then incubated in ALDH assay buffer containing the ALDH substrate (BAAA, 1 μM) for 30 min at 37°C. As a negative control, cells were stained under identical conditions in the presence of diethylaminobenzaldehyde (DEAB, 15 μM), a specific ALDH inhibitor. FACS Aria II cell sorter (BD Biosciences) was used to measure the ALDH-positive cell population.

### Statistical analysis

Data are representative of at least three different experiments. Significance of differences was determined by Student t-test analyses or by two-way ANOVA with Bonferroni's post hoc test (tumor xenograft experiments).

## Author Contributions

H.H. and J.Y. supervised the project and designed the study. H.H. and N.G. performed a most of the experimental work. M.T., N.H. and Y.K. performed some of the experiments. K.I. and K.N. synthesized the compound. H.H., N.G., Y.N. and J.Y. wrote the manuscript. All authors reviewed the manuscript.

## Supplementary Material

Supplementary Information Supplementary Table&Figures

## References

[b1] Foulkes W. D., Smith I. E. & Reis-Filho J. S. Triple-negative breast cancer. N. Engl. J. Med. 363, 1938–1948 (2010). 21067385 10.1056/NEJMra1001389

[b2] Perou C. M. *et al.* Molecular portraits of human breast tumours. Nature 406, 747–752 (2000). 10963602 10.1038/35021093

[b3] Dent R. *et al.* Triple-negative breast cancer: clinical features and patterns of recurrence. Clin. Cancer Res. 13, 4429–4434 (2007). 17671126 10.1158/1078-0432.CCR-06-3045

[b4] Wicha M. S., Liu S. & Dontu G. Cancer stem cells: an old idea--a paradigm shift. Cancer Res. 66, 1883–1890; discussion 1895–1886 (2006). 16488983 10.1158/0008-5472.CAN-05-3153

[b5] Morrison B. J., Schmidt C. W., Lakhani S. R., Reynolds B. A. & Lopez J. A. Breast cancer stem cells: implications for therapy of breast cancer. Breast Cancer Res. 10, 210 (2008). 18671830 10.1186/bcr2111 PMC2575525

[b6] Xin H. *et al.* CHIP controls the sensitivity of transforming growth factor-beta signaling by modulating the basal level of Smad3 through ubiquitin-mediated degradation. J. Biol. Chem. 280, 20842–20850 (2005). 15781469 10.1074/jbc.M412275200

[b7] Kamynina E., Kauppinen K., Duan F., Muakkassa N. & Manor D. Regulation of proto-oncogenic dbl by chaperone-controlled, ubiquitin-mediated degradation. Mol. Cell Biol. 27, 1809–1822 (2007). 17178836 10.1128/MCB.01051-06 PMC1820456

[b8] Fan M., Park A. & Nephew K. P. CHIP (carboxyl terminus of Hsc70-interacting protein) promotes basal and geldanamycin-induced degradation of estrogen receptor-alpha. Mol. Endocrinol. 19, 2901–2914 (2005). 16037132 10.1210/me.2005-0111

[b9] Kajiro M. *et al.* The ubiquitin ligase CHIP acts as an upstream regulator of oncogenic pathways. Nat. Cell Biol. 11, 312–319 (2009). 19198599 10.1038/ncb1839

[b10] Poland A., Glover E. & Kende A. S. Stereospecific, high affinity binding of 2,3,7,8-tetrachlorodibenzo-p-dioxin by hepatic cytosol. Evidence that the binding species is receptor for induction of aryl hydrocarbon hydroxylase. J. Biol. Chem. 251, 4936–4946 (1976). 956169

[b11] Rowlands J. C. & Gustafsson J. A. Aryl hydrocarbon receptor-mediated signal transduction. Crit. Rev. Toxicol. 27, 109–134 (1997). 9099515 10.3109/10408449709021615

[b12] Castro-Rivera E., Wormke M. & Safe S. Estrogen and aryl hydrocarbon responsiveness of ECC-1 endometrial cancer cells. Mol. Cell Endocrinol. 150, 11–21 (1999). 10411295 10.1016/s0303-7207(99)00041-6

[b13] Wormke M., Castro-Rivera E., Chen I. & Safe S. Estrogen and aryl hydrocarbon receptor expression and crosstalk in human Ishikawa endometrial cancer cells. J. Steroid Biochem. Mol. Biol. 72, 197–207 (2000). 10822009 10.1016/s0960-0760(00)00030-3

[b14] Morrow D., Qin C., Smith R. 3rd & Safe S. Aryl hydrocarbon receptor-mediated inhibition of LNCaP prostate cancer cell growth and hormone-induced transactivation. J. Steroid Biochem. Mol. Biol. 88, 27–36 (2004). 15026081 10.1016/j.jsbmb.2003.10.005

[b15] Koliopanos A. *et al.* Increased arylhydrocarbon receptor expression offers a potential therapeutic target for pancreatic cancer. Oncogene 21, 6059–6070 (2002). 12203118 10.1038/sj.onc.1205633

[b16] Jana N. R. *et al.* Comparative effects of 2,3,7,8-tetrachlorodibenzo-p-dioxin on MCF-7, RL95-2, and LNCaP cells: role of target steroid hormones in cellular responsiveness to CYP1A1 induction. Mol. Cell. Biol. Res. Commun. 4, 174–180 (2000). 11281733 10.1006/mcbr.2001.0275

[b17] McDougal A., Wormke M., Calvin J. & Safe S. Tamoxifen-induced antitumorigenic/antiestrogenic action synergized by a selective aryl hydrocarbon receptor modulator. Cancer Res. 61, 3902–3907 (2001). 11358803

[b18] Callero M. A. & Loaiza-Perez A. I. The role of aryl hydrocarbon receptor and crosstalk with estrogen receptor in response of breast cancer cells to the novel antitumor agents benzothiazoles and aminoflavone. Int. J. Breast cancer 2011, 923250 (2011). 22295239 10.4061/2011/923250 PMC3262585

[b19] Trapani V. *et al.* DNA damage and cell cycle arrest induced by 2-(4-amino-3-methylphenyl)-5-fluorobenzothiazole (5F 203, NSC 703786) is attenuated in aryl hydrocarbon receptor deficient MCF-7 cells. Br. J. Cancer 88, 599–605 (2003). 12592376 10.1038/sj.bjc.6600722 PMC2377159

[b20] Loaiza-Perez A. I. *et al.* Aryl hydrocarbon receptor mediates sensitivity of MCF-7 breast cancer cells to antitumor agent 2-(4-amino-3-methylphenyl) benzothiazole. Mol. Pharmacol. 61, 13–19 (2002). 11752201 10.1124/mol.61.1.13

[b21] Bradshaw T. D. *et al.* Preclinical evaluation of amino acid prodrugs of novel antitumor 2-(4-amino-3-methylphenyl)benzothiazoles. Mol. Cancer Ther. 1, 239–246 (2002). 12467219

[b22] Safe S., Qin C. & McDougal A. Development of selective aryl hydrocarbon receptor modulators for treatment of breast cancer. Expert Opin. Investig. Drugs 8, 1385–1396 (1999). 10.1517/13543784.8.9.1385 15992156

[b23] Wang X. *et al.* Comparative properties of the nuclear aryl hydrocarbon (Ah) receptor complex from several human cell lines. Eur. J. Pharmacol. 293, 191–205 (1995). 8666036 10.1016/s0922-4106(05)80044-6

[b24] Wang W. L., Porter W., Burghardt R. & Safe S. H. Mechanism of inhibition of MDA-MB-468 breast cancer cell growth by 2,3,7,8-tetrachlorodibenzo-p-dioxin. Carcinogenesis 18, 925–933 (1997). 9163677 10.1093/carcin/18.5.925

[b25] Patani N., Jiang W., Newbold R. & Mokbel K. Prognostic implications of carboxyl-terminus of Hsc70 interacting protein and lysyl-oxidase expression in human breast cancer. J. Carcinog. 9, 9 (2010). 21139993 10.4103/1477-3163.72505 PMC2997236

[b26] Tsuchiya M. *et al.* Ubiquitin ligase CHIP suppresses cancer stem cell properties in a population of breast cancer cells. Biochem. Biophys. Res. Commun. 452, 928–932 (2014). 25234599 10.1016/j.bbrc.2014.09.011

[b27] Prud'homme G. J. *et al.* Breast cancer stem-like cells are inhibited by a non-toxic aryl hydrocarbon receptor agonist. PLoS One 5, e13831 (2010). 21072210 10.1371/journal.pone.0013831 PMC2972222

[b28] Wormke M., Stoner M., Saville B. & Safe S. Crosstalk between estrogen receptor alpha and the aryl hydrocarbon receptor in breast cancer cells involves unidirectional activation of proteasomes. FEBS Lett. 478, 109–112 (2000). 10922479 10.1016/s0014-5793(00)01830-5

[b29] Wormke M. *et al.* The aryl hydrocarbon receptor mediates degradation of estrogen receptor alpha through activation of proteasomes. Mol. Cell Biol. 23, 1843–1855 (2003). 12612060 10.1128/MCB.23.6.1843-1855.2003 PMC149455

[b30] Wang J., Guo L. P., Chen L. Z., Zeng Y. X. & Lu S. H. Identification of cancer stem cell-like side population cells in human nasopharyngeal carcinoma cell line. Cancer Res. 67, 3716–3724 (2007). 17440084 10.1158/0008-5472.CAN-06-4343

[b31] Yu F. *et al.* Kruppel-like factor 4 (KLF4) is required for maintenance of breast cancer stem cells and for cell migration and invasion. Oncogene 30, 2161–2172 (2011). 21242971 10.1038/onc.2010.591 PMC3088782

[b32] Dontu G. *et al.* Role of Notch signaling in cell-fate determination of human mammary stem/progenitor cells. Breast Cancer Res. 6, R605–615 (2004). 15535842 10.1186/bcr920 PMC1064073

[b33] Harrison H. *et al.* Regulation of breast cancer stem cell activity by signaling through the Notch4 receptor. Cancer Res. 70, 709–718 (2010). 20068161 10.1158/0008-5472.CAN-09-1681 PMC3442245

[b34] Ito I. *et al.* A nonclassical vitamin D receptor pathway suppresses renal fibrosis. J. Clin. Invest. 123, 4579–4594 (2013). 24135137 10.1172/JCI67804 PMC3809783

[b35] Hirata N., Sekino Y. & Kanda Y. Nicotine increases cancer stem cell population in MCF-7 cells. Biochem. Biophys. Res. Commun. 403, 138–143 (2010). 21055391 10.1016/j.bbrc.2010.10.134

