# Supplementary material for: 2-(4-Hydroxy-3-methoxyphenyl)-benzothiazole suppresses tumor progression and metastatic potential of breast cancer cells by inducing ubiquitin ligase CHIP
Source: Sci Rep. 2014 Nov 18;4:7095. doi: 10.1038/srep07095 (PMC4235287; doi:10.1038/srep07095)
Supplement: Supplementary Information — Supplementary Table&Figures [file srep07095-s1.pdf]

2-(4-Hydroxy-3-methoxyphenyl)-benzothiazole suppresses tumor progression and metastatic potential of breast cancer cells by inducing ubiquitin ligase CHIP

Hiromi Hiyoshi, Natsuka Goto, Mai Tsuchiya, Keisuke Iida, Yuka Nakajima, Naoya Hirata, Yasunari Kanda, Kazuo Nagasawa, Junn Yanagisawa.

Supplementary Table S1. Primer sequences for real-time RT-PCR

		Forward primer/reverse primer
CHIP	forward	5'-GCAGCGTGTGGGTCATTTT-3
	reverse	5'-TAGTCCTCCACCCAGCCATT-3
Cyp1a1	forward	5'-TGGATGAGAACGCCAATGTC-3'
	reverse	5'-TGGGTTGACCCATAGCTTCT-3'
Hes1	forward	5’-AGCGGGCGCAGATGAC-3’
	reverse	5’-CGTTCATGCACTCGCTGAA-3’
Klf-4	forward	5'-GGGAGAAGACACTGCGTCA-3'
	reverse	5'-GGAAGCACTGGGGGAAGT-3'
HPRT	forward	5'-TTCCTTGGTCAGGCAGTATAATCC-3'
	reverse	5'-AGTCTGGCTTATATCCAACACTTCG-3'
GAPDH	forward	5'-ATCGTCCACCGCAAATGCTTCTA-3'
	reverse	5'-AGCCATGCCAATCTCATCTTGTT-3'
mβ-actin	forward	5’-CCCCATTGAACATGGCATTG-3’
	reverse	5’-ACGACCAGAGGCATACAGG-3’

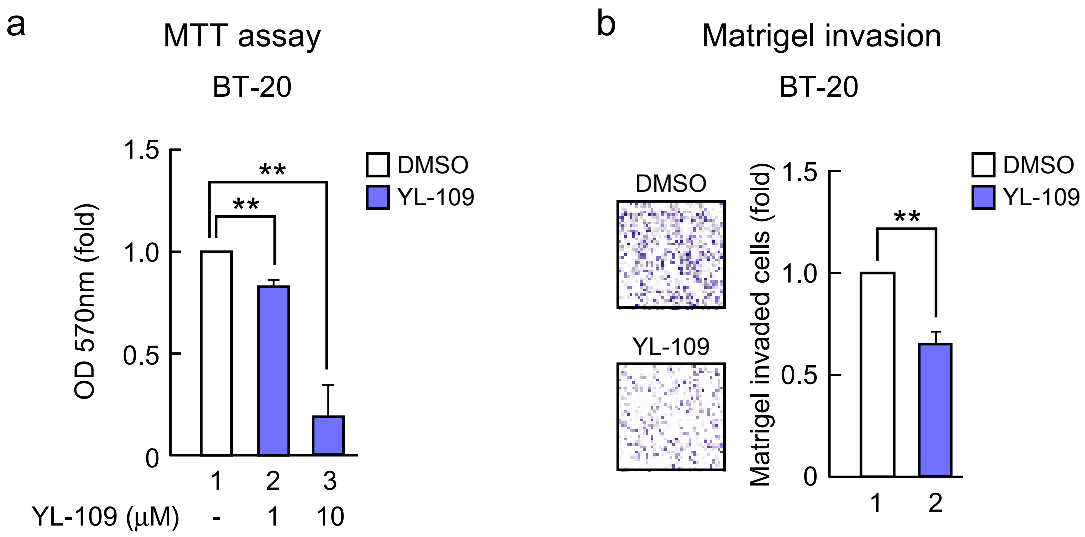

Supplementary Figure S1. YL-109 also inhibits cell proliferation and invasiveness in BT-20 cells.

(**a**) Effects of YL-109 on cell proliferation in BT-20 cells. BT-20 cells were cultured with DMSO or YL-109 (1 or 10 μM) for 96 h. The cell viability was measured by MTT assays. (**b**) Effects of YL-109 on cell invasiveness in BT-20 cells. BT-20 cells were seeded onto filters with an 8 μm pore size in Matrigel matrix-coated upper chambers in the absence or presence of YL-109 (1 μM). ** indicates $p<0.01$ by student's T test vs. DMSO-treated cells.

Figure 3b

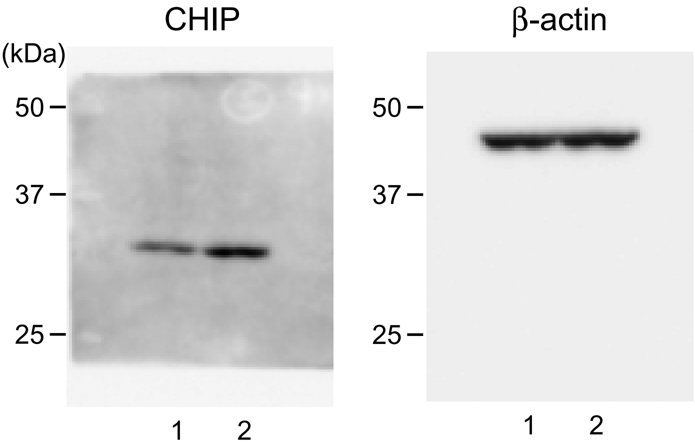

Figure 3c

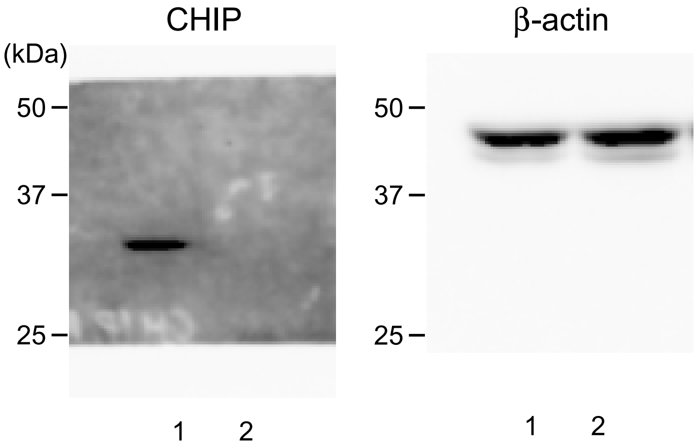

Figure 4b

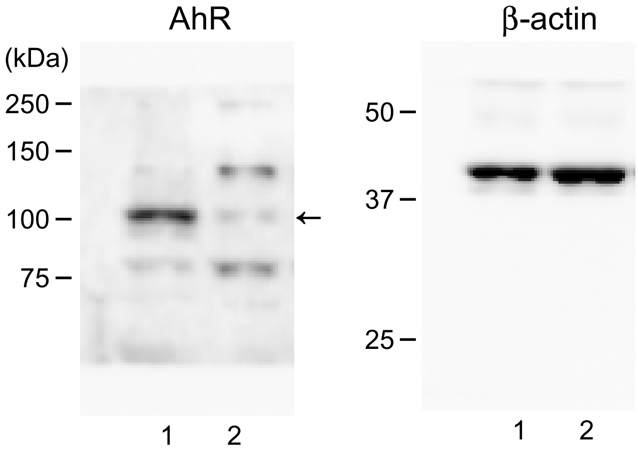

Supplementary Figure S2. Full length images of Western blots from all figures.
